# Self-Reported Environmental Tobacco Smoke Exposure and Avoidance Compared with Cotinine Confirmed Tobacco Smoke Exposure among Pregnant Women and Their Infants

**DOI:** 10.3390/ijerph15050871

**Published:** 2018-04-27

**Authors:** Adam Gregory Gavarkovs, Patricia Markham Risica, Donna R. Parker, Ernestine Jennings, Jennifer Mello, Maureen Phipps

**Affiliations:** 1 Department of Behavioral and Social Sciences, Brown School of Public Health, Brown University, Providence, RI 02912, USA; adam_gavarkovs@g.harvard.edu; 2 Department of Epidemiology, Brown School of Public Health, Brown University, Providence, RI 02912, USA; Donna_Parker@brown.edu (D.R.P.); Mphipps@wihri.org (M.P.); 3 Center for Health Equity Research, Brown School of Public Health, Brown University, Providence, RI 02912, USA; Jennifer_mello@brown.edu; 4 Memorial Hospital of RI, Center of Primary Care and Prevention, Pawtucket, RI 02904, USA; 5 Department of Family Medicine, Warren Alpert Medical School of Brown University, Providence, RI 02912, USA; 6 Center for Behavioral and Preventive Medicine, The Miriam Hospital, Providence, RI 02906, USA; ernestine_jennings@brown.edu; 7 Department of Psychiatry and Human Behavior, Warren Alpert Medical School of Brown University, Providence, RI 02912, USA; 8 Department of Obstetrics & Gynecology, Warren Alpert Medical School of Brown University, Providence, RI 02912, USA; 9 Women & Infants Hospital of Rhode Island, Providence, RI 02905, USA

**Keywords:** environmental tobacco exposure, pregnancy, infancy, measurement

## Abstract

Background: Exposure to environmental tobacco smoke (ETS) presents substantial health risks for pregnant women and newborn infants. Measurements of ETS include invasive and expensive biochemical tests, as well as less invasive and lower-cost, self-reported exposure and avoidance measures. Better understanding of self-report measures will help to select ETS assessments for evaluation. Methods: This analysis was conducted within the context of a tailored video intervention to reduce tobacco smoking and ETS exposure during pregnancy and after delivery in the control group sample of 147 nonsmoking women. Measurements of salivary cotinine concentration, self-reported ETS exposure, and avoidance behaviors were captured at 32 weeks’ gestation and 6 months postpartum. Results: Salivary cotinine concentration was significantly related to ETS avoidance among pregnant nonsmokers at 32 weeks’ gestation, but not ETS exposure. At 6 months postpartum, both the reported ETS exposure of the infant and maternal avoidance behaviors to reduce her infant’s exposure were associated with the infant’s salivary cotinine concentration. At 32 weeks’ gestation and 6 months postpartum, avoidance behaviors decreased as exposure increased. Discussion: This study suggests that for nonsmoking women during pregnancy, reports of tobacco smoke avoidance are more valid than reports of exposure. After delivery, self-reported ETS exposure or avoidance are associated with each other and the biochemical measurement of salivary cotinine. These results provide researchers and clinicians with evidence to support the inclusion of avoidance behaviors in the selection of ETS measures.

## 1. Introduction

Environmental tobacco smoke (ETS) exposure, defined as smoke emitted from the burning end of a cigarette or cigar or exhaled by a smoker, represents a well-established and significant health risk. The risk of adverse pregnancy and birth outcomes associated with ETS exposure include preterm birth [1,2,3,4], low birthweight [2,3,4], stillbirth, and congenital malformations [5]. Health concerns are also increased when newborns are directly exposed to ETS, including an increased risk of infections [6], impaired pulmonary function [7], sudden infant death syndrome [8,9,10], and behavioral problems later in life [11]. Furthermore, young children whose parents smoke are more likely to begin smoking, which is associated with a well-established host of health risks [12,13,14].

These health risks are particularly important given the number of pregnant women who may be smoke exposed. Previous studies that have defined ETS exposure during pregnancy as living with a smoker have found a wide range of exposures, depending on the population studied. In a large analysis of 18,297 births in the United Kingdom (UK), 13.5% of women reported smoke exposure [4], while another study of low-income African American pregnant women in New York City found exposure to be as high as 40% [15]. ETS exposure is also high in infancy. Among children under three years of age in a San Francisco convenience sample using discarded blood samples, 55.0% (*n* = 349) had detectable concentrations of cotinine, a metabolite of nicotine, with 92% of those exposed having serum cotinine concentrations over 5 ng/mL [16].

Evaluation and accurate measurement of ETS is a key component of efforts to reduce ETS exposure. Measurement is not only important in epidemiological efforts to quantify the extent of ETS exposure and identify segments of the population that might be particularly vulnerable, but also for determining the efficacy and effectiveness of ETS exposure reduction interventions.

Cotinine represents a biochemical method of quantifying ETS, which can be measured in biological fluids, including serum, saliva, and urine [17,18]. Although cotinine concentration may be an appropriate measure of ETS exposure, the burden of sample collection and the expense of collection and analysis are considerable, sometimes even prohibitive. Furthermore, collecting serum, saliva, or urine samples represents a time and resource-intensive procedure that may also be considered invasive by participants. Self-reported ETS exposure is a much less expensive and burdensome alternative. However, for a self-report measure to be useful, it must have a demonstrated relationship with biochemical measures of smoke exposure.

Several studies have examined the level of agreement between self-report measures of ETS exposure during pregnancy or infant exposure with biochemical markers, including cotinine concentration [19,20,21,22,23]. Another self-report measure, ETS avoidance, has been used in several studies as a behavioral outcome measure [24,25]. However, the relationship between a measure of ETS avoidance behaviors and a biochemical measure of exposure has not been evaluated.

The purpose of this paper is to explore the relationship between a biochemical measure of tobacco smoke exposure (salivary cotinine concentration) and two self-report methods: (1) ETS exposure and (2) ETS avoidance behaviors among nonsmoking pregnant women and their infant. This analysis will add to the literature examining the relationship between reported ETS exposure and biochemical metrics among pregnant women and young infants and add a novel exploration of the relationship between self-reported ETS avoidance and biochemical exposure.

This analysis was conducted within the context of an intervention of tailored videos and newsletters delivered to the homes of pregnant, low-income women to help them quit smoking or reduce their ETS exposure, as appropriate.

## 2. Methods

The Baby’s Breath program was an innovative, individually-tailored intervention with the goal of helping pregnant women (both smokers and nonsmokers) maximize their pregnancy outcomes and newborn’s health through reduction of exposure to ETS and cessation of smoking (as appropriate); it was evaluated as a randomized, controlled trial, the details and results of which are described elsewhere [25,26]. Recruitment for Baby’s Breath occurred between February of 2006 to June of 2009 with a final 6-month evaluation (after the final intervention components) in June of 2010. Beginning at recruitment, the intervention was delivered to women at or before 16 weeks’ gestation and extended to 26 weeks’ gestation. The measures included salivary cotinine measured at 32 weeks of pregnancy (for the pregnant woman) and 6 months postpartum (for the mother, as well as the infant), as well as self-reported ETS exposure and ETS avoidance queried at the baseline (16 weeks), 32 weeks, and 6 months. The present analysis will explore (1) the relationship between self-reported ETS exposure and avoidance with salivary cotinine among nonsmoking pregnant women, (2) the relationship between self-reported ETS avoidance and salivary cotinine in nonsmoking mothers after delivery, and (3) the relationship between infant ETS exposure and maternal avoidance as reported by their nonsmoking mother with infant salivary cotinine. The participants in the study were categorized as a smoker or nonsmoker separately at 32 weeks’ gestation and 6 months postpartum based on their response to a question on the survey administered at each evaluation point that asked, “*Do you currently smoke cigarettes, even occasionally?*” Only the participants from the control group (receiving no intervention) were included in these analyses to avoid any biases or changes due to the intervention. Also, only the participants with salivary cotinine ≥10 μg/mL, which is a cutoff associated with more exposure than from only environmental sources, were included.

**Biochemical ETS Exposure.** Saliva was analyzed by Salimetrics Inc., State College, PA, USA. The primary analytic method used to assess salivary cotinine concentration was liquid chromatography/mass spectrometry identification and quantification.

**ETS Exposure.** ETS exposure was captured using the self-reported TOTS measure, adapted from the Healthy Tots Project [27], which asks about five different settings where a pregnant woman/mother may be exposed to ETS. A zero on this measure represents no self-reported exposure in any setting, while a score of one through five represents the number of sites of exposure, including the workplace, a friend’s house, and a relative’s house. During pregnancy, the TOTS measure captured the self-reported exposure of the pregnant woman, whereas the 6-month postpartum measurement captured the exposure of the infant as reported by the mother. Self-reported ETS exposure with respect to the mother was not assessed at 6 months postpartum. For these analyses, the TOTS measure was collapsed into 3 categories due to unequal distributions across both time points: (1) no exposure; (2) exposure in one setting; and (3) exposure in two or more settings.

**Self-reported ETS Avoidance.** ETS avoidance was measured using the Martinelli Scale from *Avoidance of Environmental Tobacco Smoke* [28]. The Martinelli scale asked about ways in which ETS could be avoided, including items such as permitting smoking in the mother’s home and car, staying around someone who lights up, associating with smokers, and remaining in a smoking section of a restaurant. For example, one question asked, “If your friends are in a designated smoking area to smoke cigarettes, YOU will join them rather than be alone”. The respondents indicated their level of agreement with each statement on a four-point Likert scale ranging from “Almost never true” to “Almost always true”. An average of the responses for each item produced a composite score to be used in the analysis, creating an index ranging from one to four, with higher values indicating more avoidance of ETS. The questionnaire was validated in a sample of mothers (*M*_age_ = 36) and yielded an internal consistency of 0.81 [28]. At 6 weeks postpartum, the measure was adapted for the current study to capture the behaviors of the mother to help her infant avoid smoke exposure. At this time period, both the adapted and original versions of the measure were used.

Dependent variables of salivary cotinine and ETS avoidance (Martinelli Scale) were designated in two separate models with the independent variable of ETS exposure (TOTS). Analysis using an ANOVA model found age to be significantly related to salivary cotinine concentration at 32 weeks, where age was directly associated with higher concentration levels (*B* = 4.63, *F* = 22.61, *p* < 0.0001). Age was therefore, included in all models. Additionally, due to non-normal distribution of the values, salivary cotinine concentration was square root transformed (and back-transformed for the purposes of reporting results). Data are presented as back-transformed cotinine values. Also, a linear regression model was constructed with salivary cotinine as the dependent variable and with ETS avoidance (Martinelli Scale) as the independent variable, along with age.

## 3. Results

The characteristics of the sample at 34 weeks’ gestation (Table 1, *n* = 147) include young women (36% < 21 years of age and 38% 21–25 years of age). The biggest group of participants self-identified as Hispanic (39%), followed by non-Hispanic White (35%), and the highest proportion reported having a high school education or GED equivalent (44%). Most participants were employed either full time (27%) or part time (27%), though 41% were not employed and not a student. The largest proportion of the participants reported a low household income, with 38% reporting less than $10,000 per year and 35% reporting $10,000–$30,000. More than half of the sample reported being married, engaged, or living with a significant other (63%). The participants evaluated at 6 months postpartum are a subsample of this group. At 32 weeks’ gestation, just about a third of the respondents reported exposure in each of the categories of ETS exposure. At 6 months postpartum, 73% reported no exposure, 17% reported exposure in one setting, and 10% reported exposure in two or more settings (Table 2).

**Salivary cotinine by reported ETS exposure.** The mean salivary cotinine did not differ by TOTS categories (ETS exposure) at 32 weeks’ gestation (Table 2). At 6 months postpartum, the TOTS measure of ETS exposure was significantly associated with the infant’s salivary cotinine concentration (*F* = 5.70, *p* < 0.05). The salivary cotinine observed for the participants reporting two or more exposure settings (3.2 ng/mL) was significantly greater than for those reporting exposure in one setting (1.4 ng/mL) of exposure and no settings of exposure (1.3 ng/mL, *p* < 0.05).

**ETS avoidance by level of ETS exposure.** The mean ETS avoidance score was inversely associated with level of ETS exposure at both 32 weeks’ gestation and 6 months postpartum (Table 2). At 32 weeks’ gestation, ETS avoidance for those reporting exposure in two or more settings (2.9) was significantly lower than for those reporting exposure at one setting (3.2) and those reporting exposure at no settings (3.3, *p* < 0.05). Similarly, at 6 months postpartum, the ETS avoidance score was lower for women reporting ETS exposure at two or more settings (3.4) compared with women reporting exposure at one setting (3.7) or no settings (3.8, *p* < 0.05).

**Salivary cotinine and reported ETS avoidance.** A higher score on the Martinelli Scale was significantly associated with a lower salivary cotinine concentration at every time point (Table 3, *p* < 0.05). At 6 months postpartum, higher ETS avoidance for the infant and for the mother were significantly related to lower salivary infant and maternal salivary cotinine, respectively (*p* < 0.05 each). While each model showed statistical significance, the relationship of ETS avoidance for the infant explained a larger amount of the variance in salivary cotinine (*r*^2^ = 0.13) compared with maternal avoidance for herself and maternal salivary cotinine at 32 weeks’ gestation and at 6 months postpartum (*r*^2^ = 0.03 for both models).

## 4. Discussion

The results of this study demonstrate that ETS avoidance during pregnancy and later for the baby is negatively associated with salivary cotinine concentration. Self-reported ETS exposure is associated with salivary cotinine only after delivery, but not during pregnancy. Also, self-reported exposure and avoidance are associated with each other, though avoidance explains little of the variance in exposure.

Previous literature examining the relationship between self-reported ETS exposure and biochemical smoke exposure has been mixed with some studies indicating high levels of sensitivity of the self-reported measures [22,29], where others have found weak or nonexistent relationships, particularly among pregnant women where underreporting may be present [19,23,30]. However, much of the existing research evaluating ETS exposure by self-report has classified women or infants as smoke exposed based on their cohabitation with a smoker [4,15] or has evaluated smoke exposure in two settings: at home and at work [22,29]. This study expands the literature by exploring the relationship between cotinine and reported exposure in five settings, including the home and workplace, but also settings like a friend or relative’s house. As municipal, state, and nation-wide bans of indoor tobacco smoking in workplaces have been implemented in many developed countries over recent decades, the workplace may not be a prominent source of exposure as it once was [22]. Assessing exposure only at home and in the workplace does not reflect the current contexts in which pregnant women and their newborns may be smoke exposed. The smoking status of family, friends, and non-family childcare providers may impact a pregnant woman’s or infant’s risk of being ETS exposed, so it is necessary to assess exposure in other physical locations. Our study found higher salivary cotinine with higher reported numbers of exposure locations, supporting the assertion that assessment of more settings than the workplace and home is beneficial. However, this analysis categorized the participants based on the number of settings of exposure, without consideration of each setting. Future research may explore further the relative contribution of each unique setting to overall ETS exposure to determine which settings represent the most prominent sources of exposure. The assessment of exposure to the effluent of electronic nicotine delivery systems (E-Cigarettes) has also not yet been added to assessment instruments to determine the potential nicotine exposure associated with the use of those products in the environments of pregnant women and infants.

To our knowledge, there have been no previous studies that have examined the utility of a measure of reported ETS avoidance behaviors in association with biochemical exposure. This study has demonstrated that self-reported ETS avoidance of a woman during and after pregnancy, as well as ETS avoidance while with her infant, is significantly related to biochemical exposure levels. Although the explicit purpose of this measure is not to assess ETS exposure, we found that more reported efforts to avoid ETS are associated with lower biochemical smoke exposure. As such, the Martinelli Scale, including the adapted version for avoidance on behalf of an infant or child, could serve as an effective proxy for salivary cotinine in this population.

It might seem that exposure and avoidance would measure the same issues in reciprocal concept, thereby implying that separate measures are not needed. However, these data show that these participant-reported constructs are not reciprocal, but account for only a small amount of overlap of one another. Avoidance is an active behavior, which might often be within the woman’s control, whereas exposure may not always be within her control.

There are several limitations to this study to be considered as the results are interpreted. The smoking status of each participant was established based on their response to a single survey question, “*Do you currently smoke cigarettes, even occasionally?*” Clearly, self-report is not the most accurate method to establish smoking status [17,30,31]. Inaccurate reporting may have led to misclassification in our study, thereby underestimating the strength of the relationships between self-reported exposure and avoidance with a biochemical measure. However, limiting the analysis to women only with cotinine ≤10 μg/mL eliminated the participants with a cotinine level that indicated higher smoke exposure than only from environmental sources.

Another limitation to this study may have been the fact that it was performed within the context of a tailored smoking cessation and ETS exposure reduction intervention, though only the participants who received no material related to the dangers of ETS and ways to reduce their exposure were included.

However, these limitations must be placed into the context of the overall positive attributes of this work. This study collected prospective data of nonsmoking women during and after pregnancy using multiple measurement tools that are compared to indicate the relative utility of self-report versus biochemical measurement.

## 5. Conclusions

The purpose of this study was to examine the relationship between a measure of reported ETS exposure, a measure of reported ETS avoidance behaviors, and a biochemical measure of ETS exposure among nonsmoking pregnant women and young infants. Both self-report measures are related to salivary cotinine concentrations among women before and after delivery and their infants. Although cotinine concentration is a valid measure of ETS exposure, cost or inconvenience may prohibit its use in some research settings. Our results suggest that a self-reported measure of ETS exposure and ETS avoidance behaviors may be an acceptable substitute to assess ETS exposure in women before and after delivery, as well as their infants.

## Figures and Tables

**Table 1:** Demographic Characteristics of the 34-Week Sample (*n* = 147).

Variable	Category	*n* (%)
Age	Less than 21	53 (36.1)
	21–25	56 (38.1)
	26–35	35 (23.8)
	36+	3 (2.0)
Race/Ethnicity	White Non-Hispanic	52 (35.4)
	Black Non-Hispanic	16 (10.9)
	Hispanic	58 (39.5)
	Other/More than one/Unclear	21 (14.3)
Education	<8th grade/Some HS	44 (30.0)
	HS/GED	64 (43.5)
	At least Some Tech/Some College	39 (26.5)
Employment status	Employed Full Time	29 (26.9)
	Employed Part Time	29 (26.9)
	Not Employed	44 (40.7)
	Student	3 (2.8)
	Other	3 (2.8)
	Missing	39 (26.5)
Income	≤$10,000	56 (38.1)
	$10,000–$30,000	51 (34.7)
	$30,000+	24 (16.3)
	Don’t Know	16 (10.9)
Marital status	Never Married	51 (34.9)
	Married/Engaged/Living with Sig Other	92 (63.0)
	Divorced/Separated	3 (2.1)

**Table 2:** Frequency and percentage of the participants, the mean and standard deviation of salivary cotinine, and the Martinelli scores of non-smoking control group participants with salivary cotinine ≤10 μg/mL by reported exposure (TOTS) at 32 weeks’ gestation and 6 months postpartum.

	32 Weeks’ Gestation			6 Months Postpartum (Infant)		
*N*	*n* = 147	Mean (SD)		*n* = 120	Mean (SD)	
TOTS Scale (Exposure Settings)		Salivary Cotinine	Martinelli Score		Salivary Cotinine	Martinelli Score
Overall	% (*n*)	0.6 (0.3)	3.1 (0.5)	% (*n*)	1.5 (0.5)	3.8 (0.3)
0 (no settings)	37.0 (54)	0.5 (0.2)	3.3 (0.4) ^c^	73.3 (88)	1.3 (0.5) ^c^	3.8 (0.3) ^c^
1 (one setting)	30.8 (45)	0.7 (0.4)	3.2 (0.5) ^c^	16.7 (20)	1.4 (0.6) ^c^	3.7 (0.3) ^c^
2+ (two or more settings)	31.2 (47)	0.7 (0.3)	2.9 (0.5) ^a,b^	10.0 (12)	3.2 (0.7) ^a,b^	3.4 (0.6) ^a,b^
	*F*	0.93	11.44 *		5.70 *	6.05 *
	*r* ^2^	0.02	0.14		0.09	0.11

* indicates *p* < 0.05 overall difference in salivary cotinine between TOTS groups. Lettered markers indicate pairwise significance between groups as follows: ^a^ = different from “0 settings” group; ^b^ = different from “1 setting group”; and ^c^ = different from “2+ settings” group.

**Table 3:** Association of the mean salivary cotinine with environmental tobacco smoke (ETS) avoidance (Martinelli Scale) in regression models by timing of measure.

	32 Weeks’ Gestation	6 Months Postpartum (Infant)	6 Months Postpartum (Mother)
*N*	147	120	100
*B*	−0.16 *	−0.15 *	−0.14 *
*r* ^2^	0.03 *	0.13 *	0.03 *

* indicates *p* < 0.05 overall association between the salivary cotinine and the Martinelli Scale.
